# The Yeast eIF2 Kinase Gcn2 Facilitates H_2_O_2_-Mediated Feedback Inhibition of Both Protein Synthesis and Endoplasmic Reticulum Oxidative Folding during Recombinant Protein Production

**DOI:** 10.1128/AEM.00301-21

**Published:** 2021-07-13

**Authors:** Veronica Gast, Kate Campbell, Cecilia Picazo, Martin Engqvist, Verena Siewers, Mikael Molin

**Affiliations:** a Department of Biology and Biological Engineering, Chalmers University of Technology, Gothenburg, Sweden; b Novo Nordisk Foundation Center for Biosustainability, Chalmers University of Technology, Gothenburg, Sweden; c Institute for Integrative Systems Biology (I2SysBio), University of Valencia-CSIC, Paterna, Spain; Nanjing Agricultural University

**Keywords:** recombinant protein production, heterologous protein production, H_2_O_2_, hydrogen peroxide, protein kinase Gcn2, Gcn4, ER stress, oxidative stress, translational control

## Abstract

Recombinant protein production is a known source of oxidative stress. However, knowledge of which reactive oxygen species are involved or the specific growth phase in which stress occurs remains lacking. Using modern, hypersensitive genetic H_2_O_2_-specific probes, microcultivation, and continuous measurements in batch culture, we observed H_2_O_2_ accumulation during and following the diauxic shift in engineered Saccharomyces cerevisiae, correlating with peak α-amylase production. In agreement with previous studies supporting a role of the translation initiation factor kinase Gcn2 in the response to H_2_O_2_, we find that Gcn2-dependent phosphorylation of eIF2α increases alongside translational attenuation in strains engineered to produce large amounts of α-amylase. Gcn2 removal significantly improved α-amylase production in two previously optimized high-producing strains but not in the wild type. Gcn2 deficiency furthermore reduced intracellular H_2_O_2_ levels and the Hac1 splicing ratio, while expression of antioxidants and the endoplasmic reticulum (ER) disulfide isomerase *PDI1* increased. These results suggest protein synthesis and ER oxidative folding are coupled and subject to feedback inhibition by H_2_O_2_.

**IMPORTANCE** Recombinant protein production is a multibillion dollar industry. Optimizing the productivity of host cells is, therefore, of great interest. In several hosts, oxidants are produced as an unwanted side product of recombinant protein production. The buildup of oxidants can result in intracellular stress responses that could compromise the productivity of the host cell. Here, we document a novel protein synthesis inhibitory mechanism that is activated by the buildup of a specific oxidant (H_2_O_2_) in the cytosol of yeast cells upon the production of recombinant proteins. At the center of this inhibitory mechanism lies the protein kinase Gcn2. By removing Gcn2, we observed a doubling of recombinant protein productivity in addition to reduced H_2_O_2_ levels in the cytosol. In this study, we want to raise awareness of this inhibitory mechanism in eukaryotic cells to further improve protein production and contribute to the development of novel protein-based therapeutic strategies.

## INTRODUCTION

The biotechnological role of Saccharomyces cerevisiae in the production of bread and beer has been long established. In recent decades, however, this yeast has also proven effective as a host for the production of recombinant proteins of significant pharmaceutical value (1, 2). S. cerevisiae is a successful production host predominantly due to its eukaryotic posttranslational modification machinery, its ability to secrete proteins to the media, and its robustness to harsh industrial conditions, among other traits (1, 3). Many different strategies have been shown to improve recombinant protein production and secretion in yeast (4, 5), including the engineering of transport mechanisms in the secretory pathway, increasing the expression of chaperones, and even expanding the size of the endoplasmic reticulum (ER) (6–9).

Recombinant protein production is, however, known to be a significant burden for cells, due to, for example, limited secretory capacity and protein misfolding (10). In engineered high-producing strains in particular, this burden is speculated to increase concomitantly with production levels, leading to ER stress (11, 12). To counter this and the accumulation of unfolded proteins within this organelle, two mechanisms can be activated or upregulated, the unfolded protein response (UPR) and ER-associated degradation (ERAD). The UPR in S. cerevisiae is initiated by Ire1, an ER membrane protein with active subunits both in the ER lumen and on the cytosolic side. Upon Ire1 activation by ER stress, an mRNA encoding a transcription factor, Hac1, is spliced to its active form. Hac1p subsequently moves to the nucleus and activates the expression of UPR-associated genes (13).

Besides organelle-specific stress response mechanisms, eukaryotic cells also mount a general stress response. An example of this is the phosphorylation of the α-subunit of the eIF2 translation initiator factor (eIF2α) (14), which leads to the attenuation of general translation and a reduction in protein synthesis. Mammals have a total of four kinases that can phosphorylate eIF2α in response to various stress signals, PERK, PRK, GCN2, and HRI, whereas S. cerevisiae only expresses one of these, GCN2 (14). The protein kinase Gcn2 in S. cerevisiae is mainly known as the activator for the general amino acid control (15). Upon depletion of one or multiple amino acids, this response is activated to counteract amino acid depletion. Besides reducing translation, a downstream target of Gcn2 within the general amino acid control is the transcription factor Gcn4. Gcn4 is translationally regulated and activates the expression of genes involved in the biosynthesis of amino acids, among other targets (16). However, over the years, conditions other than amino acid starvation have also been shown to activate Gcn2. As these stresses also lead to general translation attenuation, the Gcn2-mediated response has subsequently been renamed the integrated stress response (15–20).

One of the stress agents known to activate the protein kinase Gcn2 in S. cerevisiae is H_2_O_2_ (21), which, at lower levels, also may function as a signaling molecule and is a by-product of multiple biochemical reactions. Intracellular levels of H_2_O_2_ and other reactive oxygen species (ROS) are usually maintained below certain thresholds to avoid deleterious effects, such as untargeted oxidation of cellular components (DNA, lipids, and protein) and, in more extreme cases, cell death (apoptosis) (22–24). When levels of ROS do exceed this threshold, cells are known to respond by upregulating antioxidant proteins, redirecting metabolism as well as attenuating growth responses, such as the protein synthesis machinery, to regain homeostasis (25).

Oxidative phosphorylation in mitochondria and protein production in the ER can both be major sources of ROS (26, 27). Recombinant protein production has also been shown to induce both ER stress and oxidative stress (26, 28). Within the ER, oxidative stress is suggested to arise due to H_2_O_2_ production during protein folding (11, 12). H_2_O_2_ is a direct by-product of the reduction of oxygen, which occurs during disulfide bond formation, an iterative process mediated by Pdi1 and Ero1 (15). Oxidative stress subsequently limits protein secretion in both Chinese hamster ovary (CHO) cells and yeast (6, 26), with the production capacity of superproducing engineered strains most likely experiencing this limitation as well.

We hypothesize that recombinant protein production induces a negative feedback loop mediated by Gcn2, resulting in the reduction of translation and protein synthesis. In this study, we provide evidence for the production of H_2_O_2_ during recombinant protein production, using hypersensitive peroxiredoxin-based probes (29). Furthermore, by removing the H_2_O_2_-activated translational initiation factor kinase Gcn2, we were able to double recombinant α-amylase production in S. cerevisiae. We find improved recombinant protein production to also correlate with the induction of the disulfide isomerase-encoding gene *PDI1* as well as several antioxidants and reduced H_2_O_2_ levels. Based on these data, we propose a model in which protein synthesis and ER folding are coupled and subject to feedback inhibition via H_2_O_2_ and Gcn2.

## RESULTS

### Recombinant α-amylase production leads to elevated levels of H_2_O_2_ in the engineered strain B184.

Previous work has shown oxidant production to limit recombinant protein production and secretion in yeast and CHO cells, respectively (6, 26). In both of these studies, the fluorescent probes used to assess oxidant production suffered from low specificity, with their response to ROS levels being impacted by peroxidase activity as well as metal ion levels. Information on the specifics of oxidant production during protein secretion subsequently remains lacking (30). Recombinant protein productivity in batch cultivation is also speculated to differ across different growth phases. Measuring this necessitates oxidant production to be monitored continuously (6), enabling subtle changes in H_2_O_2_ to be identified during different phases of cell growth. To address this, we decided to use peroxiredoxin-linked redox-sensitive green fluorescent protein (roGFP) sensors (29) in combination with microcultivation (31), considering that peroxiredoxins are by far the most H_2_O_2_-reactive proteins in the cell (32). Microcultivation was performed in the Biolector (mp2-Labs) in 48-well “flower plates” under aerobic conditions. Upon oxidation of the sensor, a fluorescent signal excited at a wavelength of 405 nm is emitted by the sensor; upon sensor reduction, this signal is instead excited at a wavelength of 488 nm. By calculating the ratio of oxidized to reduced signal (Ox/Red ratio), we were able to compare the internal H_2_O_2_ levels in different strains. We initially started with three sensors, roGFP2-*Pf*AOP, roGFP2-*Pf*AOP^L109M^, and roGFP2-Prx1, and investigated their responses to external addition of H_2_O_2_ and dithiothreitol (DTT) (see Fig. S1 in the supplemental material) (29, 33). We found the roGFP2-Prx1 sensor Ox/Red ratio to increase upon H_2_O_2_ addition and decrease upon DTT addition, whereas both roGFP2-*Pf*AOP sensors responded mainly to DTT addition (Fig. S1). Importantly, the growth of the strains expressing the roGFP2-Prx1 sensor was also similar to the wild type (Fig. S2). Based on these results, we continued our experiments only with the roGFP2-Prx1 sensor, considering that this sensor demonstrated a high sensitivity to endogenous H_2_O_2_ levels (responded to DTT), while its signal still increased upon addition of exogenous H_2_O_2_ (Fig. S1). Within this setup, we also subtracted yeast cell autofluorescence from the fluorescent signal of the roGFP2-Prx1 sensor. This was possible due to our strains harboring the roGFP2-Prx1 sensor and the vector control plasmid, having highly similar growth profiles (Fig. S3 and S4).

**FIG 1:**
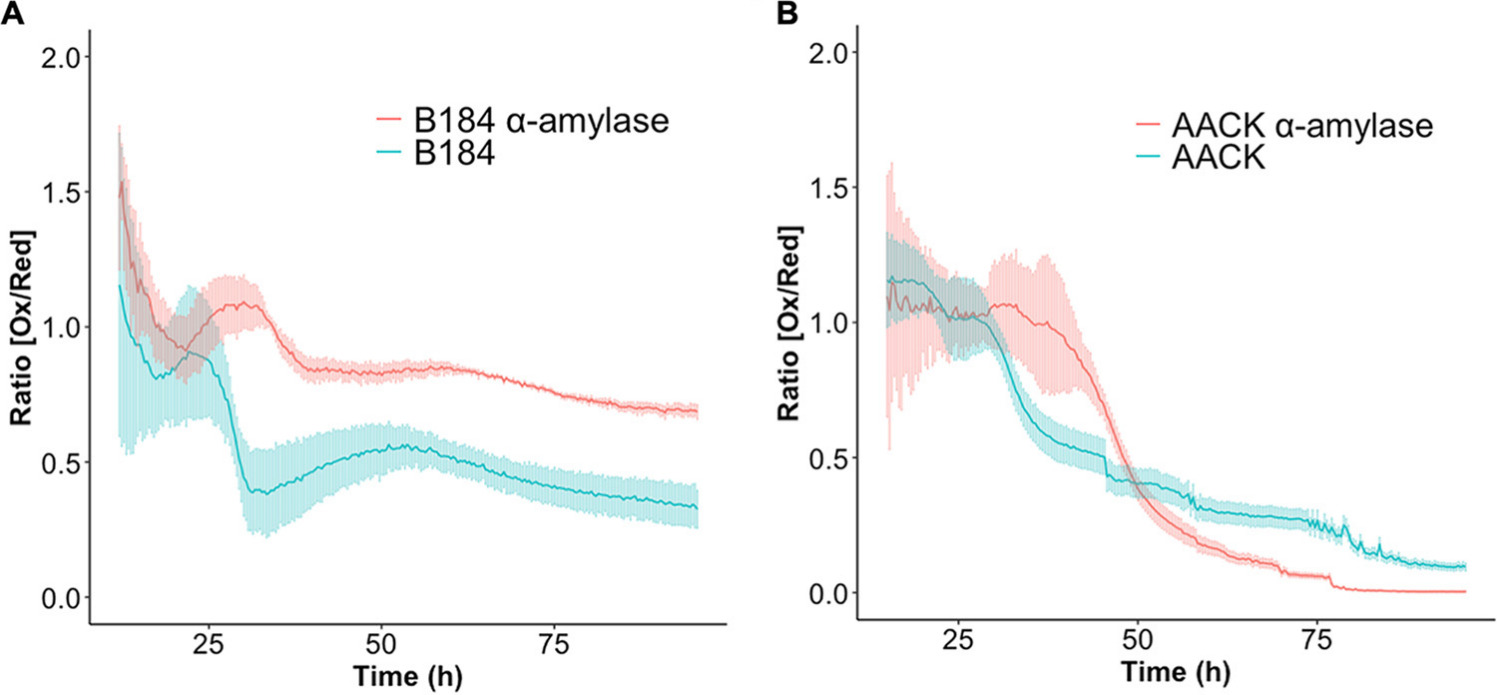
α-Amylase production leads to higher levels of intracellular H_2_O_2_ in the engineered high-level production strain B184. Ox/Red ratios over 96 h of cultivation for B184 and AACK were measured with plasmid-based roGFP-*PRX1*. (A and B) B184 (A) and AACK (B) expressing α-amylase (red) and the control without expressing α-amylase (green). The light bars represent the standard deviations from three biological replicates and two technical replicates each. The first 15 h were excluded due to insufficient signal.

Using our selected sensor, we next sought to study the impact of different levels of recombinant protein production on ROS generation. Here, we made use of B184 and AACK strains, two commonly used strains for recombinant protein production purposes. AACK is the progenitor of B184, a strain engineered by random UV mutagenesis to produce 6-fold higher α-amylase titers in batch bioreactors (34, 35). α-Amylase is used biotechnologically to release fermentable sugars from starch and is a commonly used marker protein to report on the recombinant protein production capacity in yeast cells (5, 9). We tested both strains to determine if a difference in ROS production could be observed as a consequence of their different capacities for α-amylase production. Based on the determined Ox/Red ratios, we found that recombinant α-amylase production led to increased H_2_O_2_ levels in strain B184 relative to the nonproducing strain, with this increase predominantly occurring in the later stages of growth (Fig. 1A). Since B184 demonstrates higher α-amylase production than AACK, the difference in Ox/Red ratios observed may be related to the amount of recombinant protein produced (35). In particular, we observed elevated Ox/Red ratios from around 25 h to the end of 96 h of cultivation in B184 with recombinant α-amylase production, i.e., during and following the diauxic shift (Fig. 1A). Furthermore, Ox/Red ratio levels exhibited a cell density-dependent pattern in both B184 strains, which may be related to oxygen levels and/or growth phase, as previously observed (Fig. S3) (29). In AACK, the difference with and without α-amylase production was less pronounced however with a minor peak apparent in the Ox/Red ratio between 30 h and 50 h, most likely being the result of delayed growth (Fig.1B and Fig. S4).

### The protein kinase Gcn2 is active in B184 both with and without recombinant α-amylase production.

**FIG 2:**
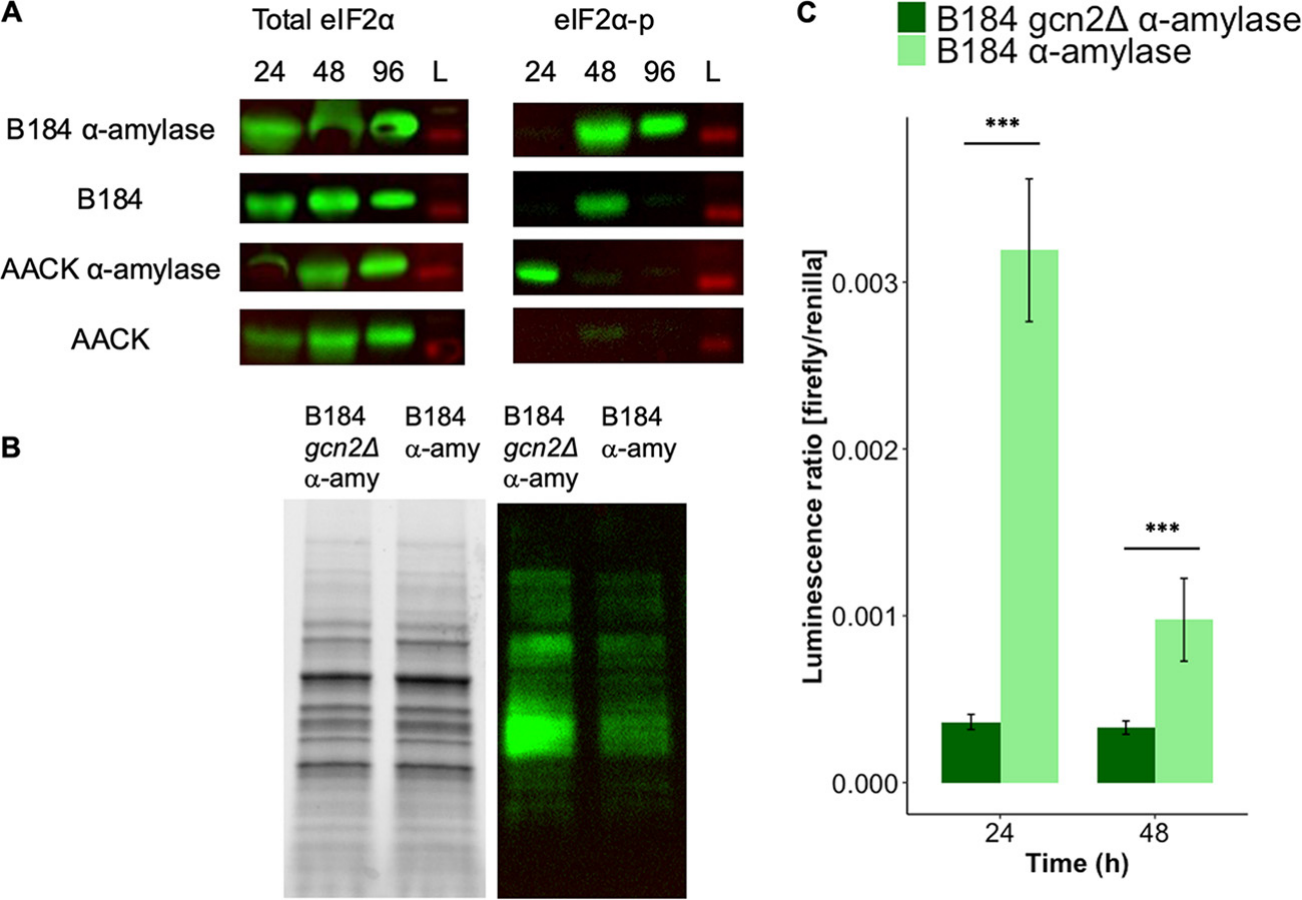
Protein kinase Gcn2 is active in the high-level production strain B184 under α-amylase-expressing conditions. Upon removal of the Gcn2 kinase, *GCN4* expression is reduced and overall translation is increased. (A) Western blot of total eIF2α and eIF2α phosphorylated during cultivation after 24, 48, and 96 h; the lane marked with L is the lane with the protein ladder. (B) Reducing SDS-PAGE and Western blot of B184 *gcn2Δ* and B184 strains while producing α-amylase with primary antibody against puromycin during the exponential growth phase (OD, 1). (C) *GCN4* expression assay based on a firefly renilla luciferase assay. The firefly luciferase gene is expressed under the control of the *GCN4* promoter, and the renilla luciferase gene is under the control of the constitutive *PGK1* promoter. The luminescence ratio of firefly luciferase/renilla luciferase represents the normalized *GCN4* expression. *GCN4* expression levels in B184 (light green) and B184 *gcn2Δ* (green) strains, both expressing α-amylase, after 24 and 48 h are shown. Significance was determined using *t* test with equal sample variance and is based on three biological replicates. *, *P* < 0.05; **, *P* < 0.01; ***, *P* < 0.005. Error bars show the standard deviations.

Previous research suggests that external H_2_O_2_ addition activates the protein kinase Gcn2 and leads to a reduction in protein synthesis (21), in part through its phosphorylation of the α subunit of the translation initiation factor (eIF2α). With the assumption that eIF2α would also respond to the increased H_2_O_2_ levels detected upon α-amylase production, we monitored Gcn2-dependent phosphorylation of eIF2α in B184 and AACK with or without α-amylase expression by immunoblotting against total and phosphorylated eIF2α. The strains were cultivated in aerated shake flasks. B184 producing recombinant α-amylase exhibited strong phosphorylation of eIF2α after 96 h (Fig. 2A), while the B184 not expressing α-amylase only showed weaker eIF2α phosphorylation at the 48-h time point (Fig. 2A). AACK showed no phosphorylation, in agreement with its redox profile (Fig. 1B and 2A). These results indicate that the phosphorylation of eIF2α in B184 after 96 h is linked to these strains’ increased capacity for α-amylase production (Fig. 2A). The phosphorylation of eIF2α was also assessed in AACK *gcn2Δ* and B184 *gcn2Δ* strains grown similarly. In AACK *gcn2Δ* and B184 *gcn2Δ* strains both with and without producing α-amylase, eIF2α remained unphosphorylated, in agreement with the idea that Gcn2 is the sole eIF2α kinase in S. cerevisiae (Fig. S5).

### The removal of the Gcn2 kinase leads to elevated rates of translation and decreased *GCN4* expression.

So far, our results indicate Gcn2 protein kinase activity in B184-producing recombinant proteins. To explore this further, we deleted *GCN2* in this strain and monitored how this would affect its best-known downstream targets, namely, genes involved in general translation and the translation of the transcription factor Gcn4. The rate of translation was measured using puromycin, a structural analog of aminoacyl-tRNAs that can be incorporated into the polypeptide chain but which prohibits further elongation (36). The strains were cultivated in aerated shake flasks. We included B184 and B184 *gcn2Δ* strains producing α-amylase. Increased levels of puromycin-bound protein could be clearly seen in the B184 *gcn2Δ* strain producing recombinant α-amylase compared to B184 producing recombinant α-amylase when *GCN2* is expressed, suggesting that a higher rate of translation can be achieved when *GCN2* is absent (Fig. 2B).

Next, we quantified the expression of *GCN4*, which, alongside the general translation rate, is regulated by Gcn2 activity. Several conditions activate Gcn2-mediated induction of *GCN4*, most of which are starvation related (17, 18, 37). Under nonstarvation conditions, *GCN4* expression is inhibited through a posttranscriptional mechanism involving four upstream open reading frames (uORFs) that are preferentially translated over the *GCN4* ORF (17, 38). In contrast, during starvation and Gcn2 activation, the low levels of ternary complexes between eIF2-GTP and the initiator tRNA-Met delay pairing with the AUG start codon sufficiently to bypass the uORFs and instead stimulate *GCN4* translation (17, 38). The expression of *GCN4* was determined using a luciferase assay with one construct expressing firefly luciferase under the control of the *GCN4* promoter and posttranscriptional regulatory regions and a control renilla luciferase under the control of a constitutive promoter (39). The cells used in this experiment were grown in aerated 24-well plates. We verified the functionality of the construct using chemically induced amino acid starvation (3-aminotriazole) (Fig. S6). The removal of the protein kinase Gcn2 in B184-producing recombinant α-amylase reduced *GCN4* expression significantly, in agreement with Gcn2 being the major activator of *GCN4* (Fig. 2C) (40). In B184 cells producing recombinant α-amylase, *GCN4* expression was visible at 24 h; however, its levels decreased at time points during which Gcn2 activity increased (from 24 h to 48 h) (Fig. 2C). Taken together, these results show that in B184 producing recombinant α-amylase, the protein kinase Gcn2 is active in reducing overall translation, whereas the expression of Gcn4, in contrast, is reduced.

### The removal of the protein kinase Gcn2 leads to an improvement of recombinant α-amylase production in two engineered production strains.

**FIG 3:**
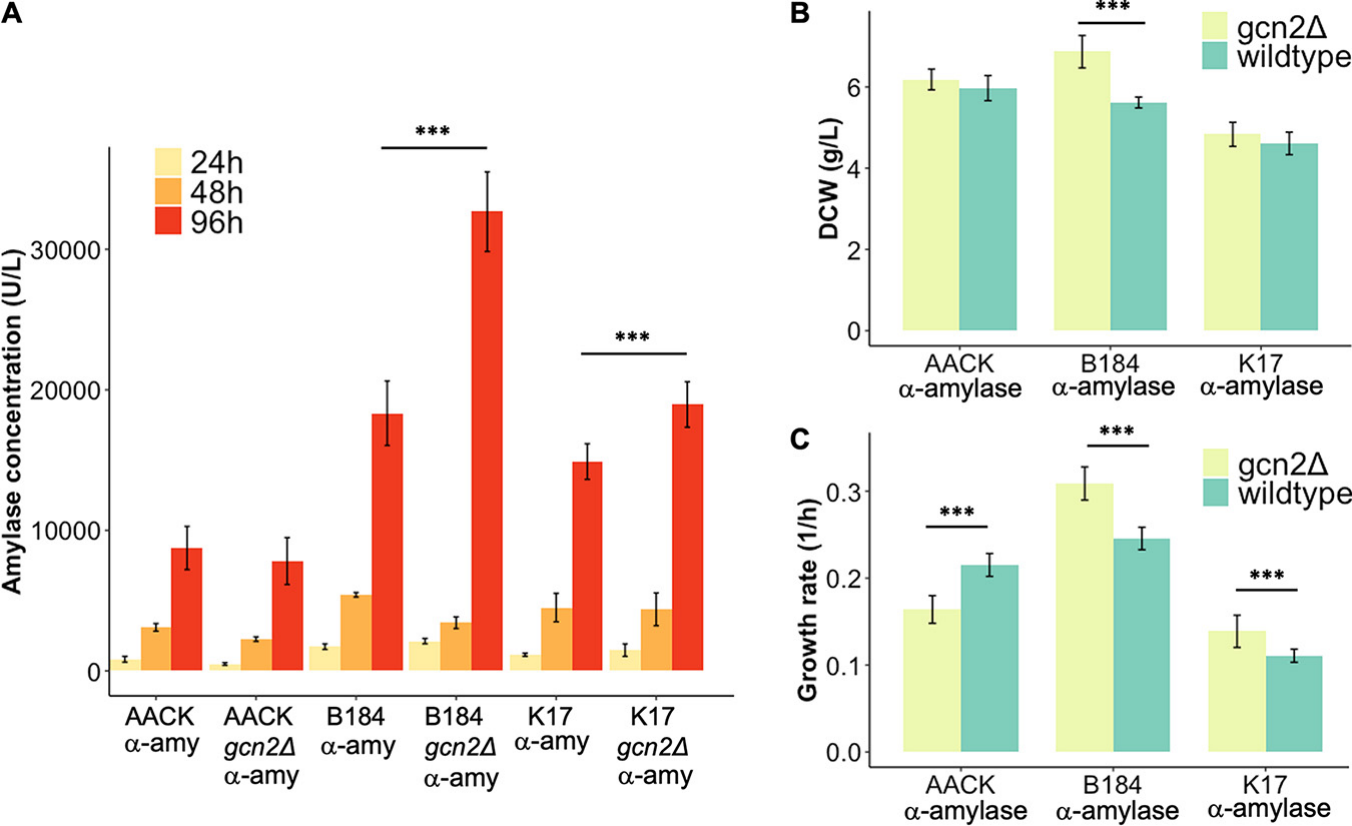
Removal of the protein kinase Gcn2 increases the α-amylase titer and improves growth parameters in two engineered high-level protein production strains. (A) α-Amylase concentration in the medium after 24 (yellow), 48 (orange), and 96 (red) h of cultivation, indicated by enzymatic assay. Data are averages from three biological replicates and two technical replicates each. Results from statistical analyses were performed for the samples at 96 h and determined based on the biological replicates only. We used the *t* test with equal sample variance. (B) Dry weight measurements after 96 h of cultivation in 24-well plates with the strains with intact *GCN2* (green) and *GCN2* removed (light green). Data presented are average values from three biological replicates and two technical replicates. (C) Exponential growth rates in 96-well plates with the strains with intact *GCN2* (green) and *GCN2* removed (light green). Data presented are average values from three biological replicates and three technical replicates. Significance was determined based on the biological replicates and technical replicas using *t* test with equal sample variance. *, *P* < 0.05; **, *P* < 0.01; ***, *P* < 0.005. Error bars show the standard deviations.

Having confirmed the activity of the protein kinase Gcn2 in repressing protein synthesis in B184, we wanted to quantify its impact on recombinant α-amylase production. We removed *GCN2* in two additional strains, AACK and K17, which is optimized for α-amylase production and secretion by targeted engineering (5). K17, like B184, is engineered to improve protein production and reaches 5-fold α-amylase titers in bioreactors compared to the AACK strain (5, 35). Using these three strains both with and without the Gcn2 kinase, we quantified the amount of α-amylase produced, selecting time points that reflected the different stages of growth. We grew the *gcn2Δ* and the control strains expressing recombinant α-amylase in aerated 24-well plates for 96 h and sampled α-amylase after 24 h, 48 h, and 96 h. These results showed that final α-amylase titers in the media increased by approximately 2-fold in B184 upon *GCN2* removal (Fig. 3A). Due to its previous engineering, B184 is already acknowledged as an efficient recombinant protein-producing strain, particularly in combination with the CPOT expression plasmid (34, 58). In comparison, for the K17 *gcn2Δ* strain, the α-amylase titer increased 30%. The removal of the protein kinase Gcn2 also turned out to have the highest impact on α-amylase production for all strains measured between 48 h and 96 h of cultivation (Fig. 3A). Finally, for AACK, the removal of the protein kinase Gcn2 had no impact on α-amylase titer at any time point during the 96 h of cultivation (Fig. 3A). In addition to α-amylase productivity, we observed a significant increase in dry weight for the B184 *gcn2Δ* strain while producing recombinant α-amylase compared to B184 *GCN2* (Fig. 3B), which agrees with this strain having a relatively higher translation rate (Fig. 2B).

Lastly, we determined the exponential growth rates for all three strains with and without *gcn2Δ.* Here, growth rates significantly increased for B184 *gcn2Δ* and K17 *gcn2Δ* strains, while a decrease was observed for the AACK *gcn2Δ* strain (Fig. 3C). Therefore, despite Gcn2 appearing to be beneficial for growth in AACK, for engineered strains wherein recombinant protein production is optimized, this protein kinase instead has a detrimental impact. This supports our previous findings that Gcn2 is more active in engineered B184 strains, most likely due to its response to increased ROS levels during amylase production (Fig. 1A and 2A and B).

### The removal of the Gcn2 kinase leads to decreased UPR activation, whereas *PDI1* expression is upregulated.

**FIG 4:**
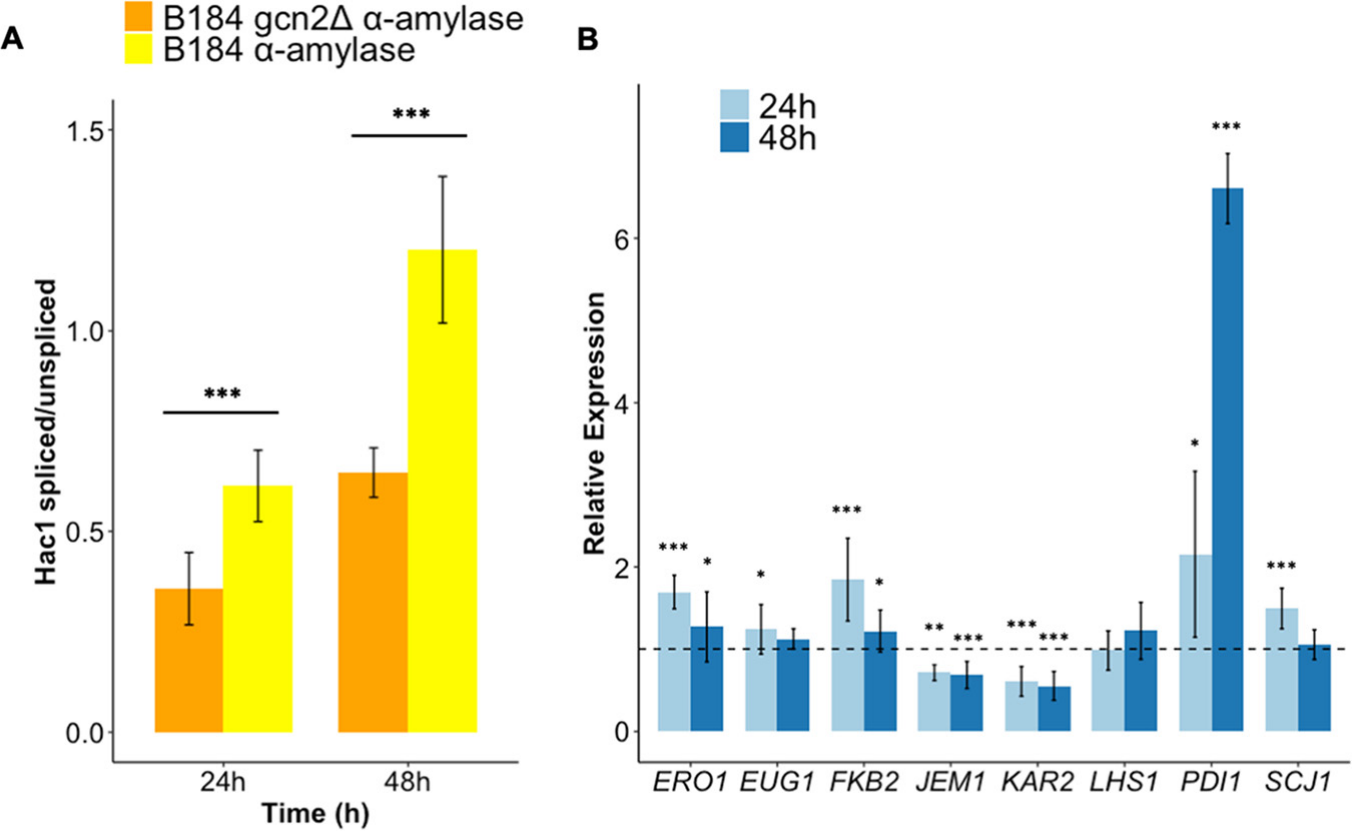
Removal of the eIF2 kinase Gcn2 reduces *HAC1* mRNA splicing, while the expression of *PDI1* is strongly increased. For all the mRNA samples, three biological replicates and three technical replicates each were used. (A) Ratio of spliced to unspliced *HAC1* mRNA. The ratio is determined per sample based on the change in threshold cycle (Δ*C_T_*) of the spliced and the Δ*C_T_* of the unspliced *HAC1* mRNA. It shows the Hac1 splicing of B184 (yellow) and B184 *gcn2Δ* (orange) strains after 24 h and 48 h. Significance was determined on the difference of the splicing ratios between the two strains using *t* test with equal sample variance. (B) Expression levels of UPR genes determined by qPCR. The data were analyzed using the ΔΔ*C_T_* method, and the data points indicate the relative expression of the genes encoding UPR target proteins in the B184 *gcn2Δ* strain compared to B184. The dashed line indicates 1. For all the genes, the mRNA was analyzed at 24 h (light blue) and at 48 h (dark blue). Significance was determined by the difference of the Δ*C_T_* per gene between the two strains. *, *P* < 0.05; **, *P* < 0.01; ***, *P* < 0.005. Error bars show the standard deviations.

To understand how *GCN2* may be linked to ROS production, we continued this study by examining the unfolded protein response (UPR) and the oxidative stress response, since these two mechanisms are intricately interconnected and have been previously linked to the control of translation (41). Cells used in quantitative PCR (qPCR) analysis were cultivated in shake flasks. The UPR in S. cerevisiae is activated by the Hac1 transcription factor, which itself is posttranscriptionally controlled by a splicing mechanism induced upon ER stress. Here, the spliced mRNA of *HAC1*, when translated into its active form, leads to it inducing the transcription of the UPR genes (13). Therefore, we measured the degree of *HAC1* mRNA splicing in B184 and B184 *gcn2Δ* strains while producing α-amylase by qPCR to decipher if the UPR was being activated. Interestingly, both B184 and B184 *gcn2Δ* strains showed an increase in the *HAC1*^spliced^-to-*HAC1*^unspliced^ mRNA ratio from 24 h to 48 h, suggesting that *HAC1* is more active in later stages of cell growth. When comparing the B184 *gcn2Δ* strain to B184, however, the *HAC1*^spliced^-to-*HAC1*^unspliced^ mRNA ratio was lower after 24 h and 48 h (Fig. 4A), suggesting that this strain experiences less ER stress.

We next selected several transcriptional Hac1 targets to check for their expression levels following *GCN2* deletion while producing α-amylase (Fig. 4B). We found that almost all genes had increased expression in the B184 *gcn2Δ* strain, even though the *HAC1*^spliced^-to-*HAC1*^unspliced^ mRNA ratio was lower (Fig. 4A). However, the expression of the UPR target genes decreased from 24 h to 48 h. The only exception was *PDI1*, the transcript of which increased 7-fold after 48 h in the B184 *gcn2Δ* strain compared to B184. The expression of *PDI1*’s counterpart in disulfide formation, *ERO1*, was only modestly increased (Fig. 4B). Thus, the higher abundance of the *PDI1* transcript in the B184 *gcn2Δ* strain seems independent of the UPR. The other known UPR target genes, *KAR2*, *JEM1*, *EUG1*, *SCJ1*, and *LHS1* (Fig. 4B), showed expression similar to that of *ERO1*, in which their expression was moderately increased in the B184 *gcn2Δ* strain after 24 h and showed similar expression in B184 and B184 *gcn2Δ* strains after 48 h. The exceptions were *KAR2* and *JEM1* (Fig. 4B). *KAR2* and *JEM1* showed a decreased transcript level, which correlates with the lower *HAC1*^spliced^-to-*HAC1*^unspliced^ mRNA ratio (Fig. 4A).

### Removal of the protein kinase Gcn2 leads to reduced H_2_O_2_ levels and an upregulation of antioxidant protein expression.

**FIG 5:**
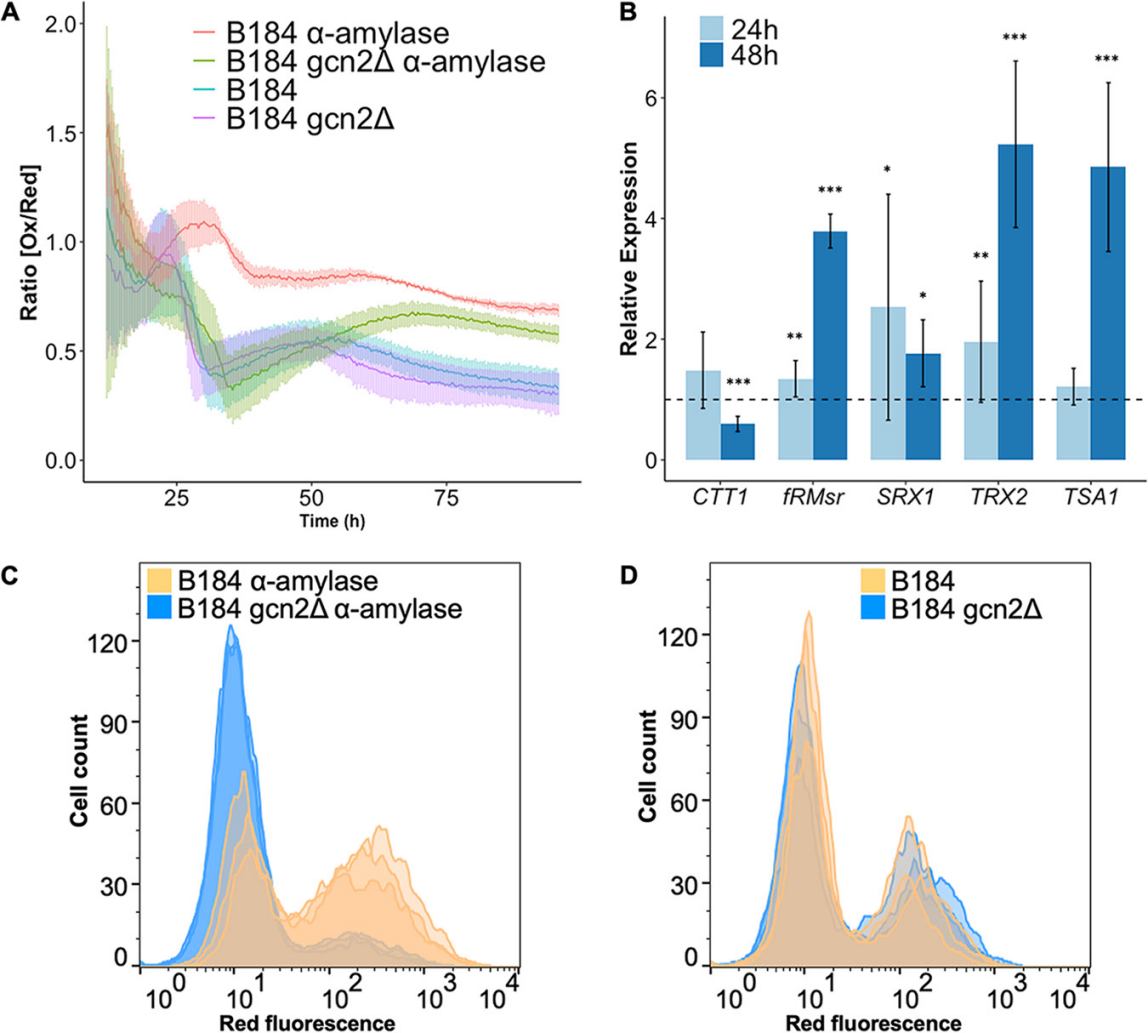
Removal of the protein kinase Gcn2 reduces H_2_O_2_ levels in B184 producing α-amylase, increases mRNA abundance of several antioxidant proteins, and improves long-term survival. (A) Ox/Red ratios over 96 h of cultivation for B184 measured with plasmid-based roGFP-*PRX1*. B184 expressing α-amylase (red), B184 *gcn2Δ* strain expressing α-amylase (green), B184 not expressing α-amylase (blue), and B184 *gcn2Δ* strain not expressing α-amylase (purple). This figure includes the same experimental data as that shown in Fig. 1A. The light bars represent the standard deviations from three biological replicates and two technical replicates each. The first 15 h were excluded due to too low a signal. (B) Expression levels of antioxidant genes determined by qPCR. The data were analyzed using the ΔΔ*C_T_* method and the data points indicate the relative expression of the genes encoding antioxidant proteins in B184 *gcn2Δ* strain compared to B184. The dashed line visualizes 1. For all the genes, the mRNA was analyzed at 24 h (light blue) and at 48 h (dark blue). Significance was determined by the difference of the Δ*C_T_* per gene between the two strains. Data are based on three biological replicates, each calculated by averaging three technical replicates. Significance was determined using *t* test with equal sample variance. *, *P* < 0.05; **, *P* < 0.01; ***, *P* < 0.005. Survival was measured with PI staining in combination with flow cytometry after 13 days of cultivation. (C and D) Flow cytometry histograms with B184 (orange) and B184 *gcn2Δ* (blue) strains expressing recombinant α-amylase (C) and without recombinant protein production (D). The images contain three biological replicates per strain.

So far, our results suggest that *GCN2* deletion reduces ER stress during α-amylase production by an unknown mechanism. Next, we assessed the impact of Gcn2 removal on H_2_O_2_ production. Using a setup similar to that in the previous experiment, we compared H_2_O_2_ levels in the B184 *gcn2Δ* strain with and without recombinant α-amylase production using the roGFP2-Prx1 sensor (Fig. 5A). Across the duration of the entire cultivation, H_2_O_2_ levels were comparatively higher in B184 engineered for recombinant α-amylase production with *GCN2* intact (Fig. 5A). In the control without recombinant α-amylase production, the removal of *GCN2* did not impact the Ox/Red ratio during the cultivation. B184 *gcn2Δ* strain producing α-amylase showed an Ox/Red ratio profile more similar to the controls, which are lower than those of the B184 strain producing α-amylase. Considering that B184 *gcn2Δ* α-amylase can achieve significantly higher amylase titers than when *GCN2* is expressed (Fig. 3A), it is possible the concomitant lower H_2_O_2_ levels we observe is reflecting increased protein production in the ER in the absence of an ER stress response being triggered by *GCN2*.

Among our B184 strains, growth profiles with the roGFP2-Prx1 sensor and the control plasmid without the sensor were comparable (Fig. S7), highlighting that the inclusion of this sensor did not introduce any confounding effects in our analysis. To evaluate to what extent the decreased H_2_O_2_ levels observed in *gcn2Δ* cells reflected altered antioxidant levels, we next determined the expression of antioxidant proteins by qPCR. Except for *CTT1*, a clear increase in relative expression levels could be seen for all antioxidant-related genes tested, especially after 48 h when comparing the B184 *gcn2Δ* strain to B184 (Fig. 5B). *SRX1*, *fRMsr*, *TRX2*, and *TSA1* all showed elevated expression levels in the B184 *gcn2Δ* strain compared to B184. The upregulation of most of the antioxidant genes we tested in the B184 *gcn2Δ* strain also correlates with this strain having lower overall levels of H_2_O_2_ (Fig. 5A). Taken together with observations in the B184 *GCN2* strain, these results suggest that the presence of the protein kinase Gcn2 reduces the wild-type oxidative stress response upon α-amylase production.

### The removal of the Gcn2 kinase increases survival in recombinant α-amylase-producing B184.

ER stress has previously been suggested to increase the levels of mitochondrially derived ROS, exerting a negative effect on cell survival (28). Therefore, we tested if the removal of *GCN2* with and without recombinant α-amylase production affected survival as a consequence of its impact on ER-regulated UPR (Fig. 4), H_2_O_2_ levels (Fig. 5A), and antioxidant gene expression (Fig. 5B) in the cell. Using propidium iodide (PI) staining in combination with flow cytometry, we could visualize and quantify the proportion of dead cells in our strain cell populations. Fluorescent subpopulations indicate living cells. The strains were cultivated in aerated shake flasks. All strains showed 100% viability during the first 96 h of cultivation (Fig. S8). After 13 days, however, the fraction of surviving cells increased in the B184 *gcn2Δ* cultures upon recombinant α-amylase production compared to B184 (Fig. 5C) but not in the control without recombinant protein production (Fig. 5D), suggesting that sustained ER stress in strains engineered to increase α-amylase production eventually affects cell survival, as is more imminently apparent in ERAD-deficient cells (28).

## DISCUSSION

This work examined the roles of oxidants on recombinant protein production in yeast. We provide evidence for the accumulation of cytosolic H_2_O_2_ in cells engineered to produce high levels of α-amylase preferentially during the diauxic shift and postdiauxic shift growth phases. These are time points during which amylase production peaks, suggesting that increased H_2_O_2_ is indeed a result of recombinant protein production (6, 26).

**FIG 6:**
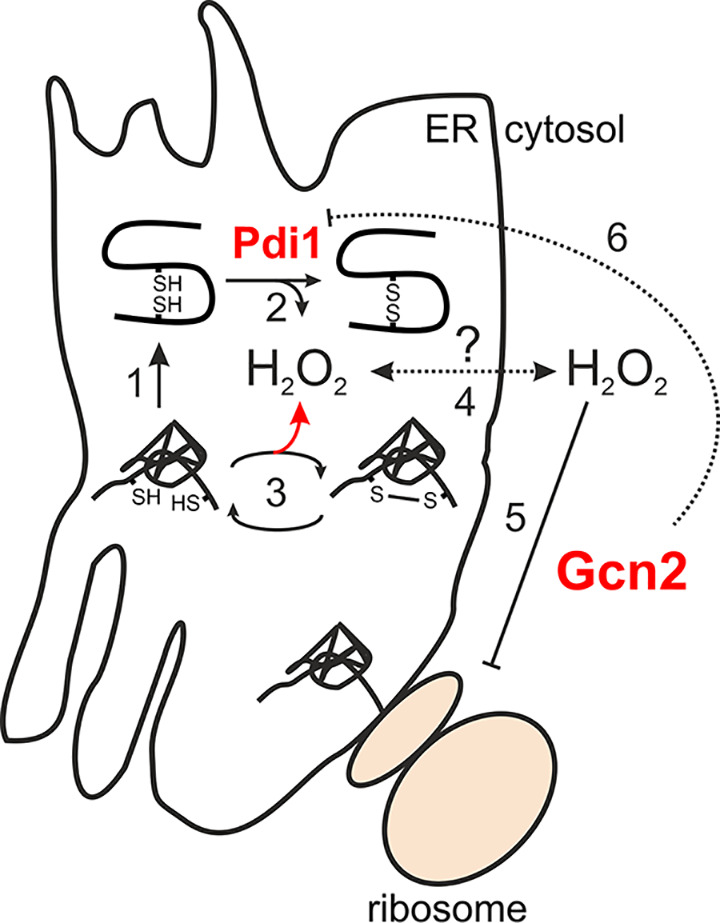
Model of mechanisms by which Gcn2 affects protein synthesis and ER oxidative folding. Recombinant protein production in the ER requires proteins secreted to be folded (1) and to form the correct disulfide bonds via Ero1/Pdi1 (2), resulting in the production of H_2_O_2_. In the case of incorrect folding and/or disulfide bond formation (3), disulfide bonds formed need to be reduced and reformed, leading to increased ER H_2_O_2_ production (red arrow). Via an unknown mechanism, recombinant protein production also leads to the accumulation of H_2_O_2_ in the cytosol (4). Cytosolic H_2_O_2_ activates the translation initiation factor (eIF2) kinase Gcn2 (5), causing the repression of protein synthesis. Through an unclear mechanism, Gcn2 also appears to exert an inhibitory effect on both antioxidant and *PDI1* expression (6), suggesting that cytosolic protein synthesis is coordinated with ER oxidative folding.

Interestingly, a recent study found that increased endogenous H_2_O_2_ levels preferentially react with cysteines in proteins of the protein synthesis machinery, potentially explaining its inhibitory effect on protein production (42). Furthermore, H_2_O_2_ has been shown to repress protein synthesis in part through activating the eIF2α kinase Gcn2 (21). In agreement with these studies, we found that the protein kinase Gcn2 was activated in engineered S. cerevisiae strains producing recombinant α-amylase, downregulating translation, and reducing α-amylase production (Fig. 2A and B and 3A). These data are consistent with a model in which cytosolic H_2_O_2_, accumulating as a result of recombinant protein production and secretion, represses cytosolic translation via the translation initiation factor (eIF2) kinase Gcn2 (Fig. 6). In support of this model, the phosphorylation of eIF2 increases in a Gcn2-dependent manner upon α-amylase production (Fig. 2A; see also Fig. S5 in the supplemental material). Furthermore, cytosolic translation is maintained to a higher degree in Gcn2-deficient cells producing amylase (Fig. 2B). Unexpectedly, however, we found that both the ER-specific UPR and oxidative stress responses were affected by the removal of *GCN2*. Whereas the Hac1 splicing ratio decreased in cells lacking Gcn2 (Fig. 4A), the antioxidant response increased (Fig. 5B), correlating with the decrease in cytosolic H_2_O_2_ observed (Fig. 5A).

### Reduction of the UPR in B184 *gcn2*Δ strain.

The UPR has previously been coupled to elevated H_2_O_2_ levels and oxidative stress. Haynes et al. observed that in ERAD-deficient cells challenged with increased levels of misfolded proteins, the removal of the UPR reduced oxidative stress and improved fitness (28). We observed a decrease in the Hac1 splicing ratio and reduced H_2_O_2_ levels upon loss of Gcn2. The level of oxidative stress has previously been thought to be the result of folding in the ER (11, 12). This is not coherent with our data, however, since we also observe increased α-amylase production upon Gcn2 removal (Fig. 3A). Besides, the UPR target genes show a variable expression pattern.

A somewhat surprising finding in this study was the rather strong induction of *PDI1* (Fig. 4B) that appears to be unrelated to the UPR. In particular, we observed an almost 7-fold induction of the *PDI1* transcript in B184 cells lacking Gcn2 (Fig. 4B). Previous studies have shown that overexpression of *PDI1* elicits a positive influence on protein production, e.g., of α-amylase (5, 34). Thus, this indirectly induced overexpression of *PDI1*, caused by the absence of protein kinase Gcn2, could be an additional explanation for the increase in α-amylase production in this strain. The strain B184 indeed carries a chromosomal duplication, leading to two copies of the *PDI1* gene in the genome, and, interestingly enough, K17, in which α-amylase production is improved upon *GCN2* removal, contains a *PDI1* overexpression cassette (5, 34). Therefore, the improved α-amylase productivity could be related to increased *PDI1* expression in K17 (Fig. 3A), observations that may explain why in AACK we do not observe improvement in α-amylase productivity. The mechanism that results in this strong induction of *PDI1* (Fig. 4B) in the absence of Gcn2 is presently unknown. Two independent large-scale transcriptomic studies, however, point out the transcriptional activator of ribosomal genes, Sfp1, as a regulator of *PDI1* (43, 44), suggesting coordination between the cytosolic protein synthesis machinery and ER-localized oxidative folding (Fig. 6).

The Hac1-mediated induction of the UPR occurs via binding to UPR elements, UPREs. Previous research has shown that there are at least three different UPREs, with the expression of associated target genes being dependent not only on Hac1 activity but also on Gcn4 expression, the downstream target of Gcn2 (45). It has also been shown that the removal of the protein kinase Gcn2 blocks the expression of UPR genes independently of *HAC1* splicing upon oxidative folding stress (45). Other studies indicate, however, that Hac1 binds independently of other factors to at least two of the UPREs (46). Specifically, *KAR2* contains the UPRE referred to as UPRE-1 in its promoter (45), and so does the promoter of *JEM1* in the strain we used. Thus, their downregulation is coherent with the reduced *HAC1* mRNA splicing observed in the B184 *gcn2Δ* strain (Fig. 4A). Based on our results, the expression of *KAR2* and *JEM1* correlates with the *HAC1* mRNA splicing ratio, indicating that the UPRE-1-mediated expression of those genes is influenced by neither Gcn2 nor Gcn4 activity.

### Removal of the Gcn2 kinase and its impact on H_2_O_2_ levels.

Interestingly, we could demonstrate that the removal of the protein kinase Gcn2 in B184 leads to a decrease in cytosolic H_2_O_2_ levels, even though α-amylase production is higher (Fig. 1A, 3A, and 5A). H_2_O_2_ is a by-product of the iterative process of forming disulfide bridges in proteins secreted via the ER (28, 47) (Fig. 6). The model protein used for this study, α-amylase, is a recombinant protein with four internal disulfide bonds and a glycosylation site. Therefore, the folding of α-amylase was expected to lead to larger amounts of oxidative stress than a less complex protein with fewer internal disulfide bonds. However, ROS accumulation has previously been suggested to increase upon the inhibition of ER glycosylation by tunicamycin as well as upon the production of a protein with significantly less complex disulfide bond formation and no glycosylation sites (48, 49). Because the formation of an incorrect disulfide bond necessitates both disulfide bond breaking and the reiteration of the H_2_O_2_-generating Ero1/Pdi1-mediated disulfide bond-forming reaction, the correct folding of the protein to be secreted is expected to reduce ER H_2_O_2_ production (Fig. 6). Thus, it was proposed that the relative rates of ER folding, including glycosylation, versus disulfide bond formation must be taken into account to explain ROS accumulation upon recombinant protein production (48). A more modern view posits that the ability of Pdi1 and Ero1 to support ER disulfide bond formation is determined by complex redox regulation of Pdi1 (and the Ero1 oxidase) via regulatory disulfide bonds (50). Furthermore, Ero1 activation is coupled to reciprocal regulation of glutathione export to the cytosol (via the ER Hsp70 Kar2), suggesting complex multilayered coordination of ER folding and disulfide bond formation (51). Interestingly, a recent investigation utilizing H_2_O_2_- and organelle-specific probes suggested that H_2_O_2_ levels in the ER are maintained mostly independently of those in the cytosol or mitochondria (52). In agreement with our data suggesting that H_2_O_2_ is originating outside the ER while still interfering with ER oxidative homeostasis, mitochondrially derived H_2_O_2_ has been shown to increase cytosolic H_2_O_2_ levels in ERAD-deficient cells challenged with increased protein secretion (28), suggesting that cytosolic H_2_O_2_ levels indeed increase under more severe ER folding stress (Fig. 6).

We find also that reduced levels of cytosolic H_2_O_2_ in cells lacking Gcn2 correlate with the upregulation of several antioxidant genes, such as *TSA1*, *TRX2*, *SRX1*, and *fRMsr* (Fig. 5A and B). Trx2 is a thioredoxin and is known to reduce cytosolic 2-Cys peroxiredoxins like Tsa1, while Srx1 is a sulfiredoxin that reactivates hyperoxidized Tsa1 (53). Interestingly, in support of the importance of Gcn2 in the antioxidant response, this protein has previously been shown to be required for high-level translation of the *SRX1* mRNA (53). Furthermore, *TSA1*, *TRX2*, and *SRX1* are all known targets of Yap1, a transcription factor that responds to elevated H_2_O_2_ levels (54–56). These genes increased expression, suggesting that Yap1 is activated in the B184 *gcn2Δ* strain while producing recombinant α-amylase. Previous work by Delic et al. showed that by overexpressing *YAP1*, the redox balance of the cytosol in a recombinant protein producing the P. pastoris strain was restored (57).

With the findings in this study, we conclude that in two strains engineered for optimized protein production, the protein kinase Gcn2 is responsible for mediating a negative feedback loop affecting both cytosolic translation and the secretory pathway. By removing this H_2_O_2_-mediated feedback loop, recombinant protein production is improved, indicating that the reduction of translation via endogenous oxidants can limit the productivity of yeast cells. This results from activation of the protein kinase Gcn2 negatively affecting several processes in the cell, including ER stress and H_2_O_2_ levels. Such findings are relevant for the engineering of production hosts for biotechnological production processes but also in basic research through the understanding of a feedback loop coordinating cytosolic protein synthesis with protein secretion.

## MATERIALS AND METHODS

### Strains and plasmids.

Three previously constructed S. cerevisiae strains were used in this study. CEN.PK 530.1CK [*MATα URA3 HIS3 LAU2 TRP1 SUC2 MAL2-8^c^ tpi1*(41-707)] is further referred to as AACK. Previous studies have engineered AACK to improve protein production, leading to two strains, B184 and K17 (5, 34). B184 is generated by UV mutagenesis, and K17 has the genotype AACK (*Δhda2 Δvps5 Δtda3 PGK1p-COG5 Δgos1*::*amdSYM-TEF1p-PDI1*). AACK, B184, and K17 additionally have a disrupted *TPI1* gene. To complement this deficiency, we use the pAlphaAmyCPOT plasmid with an expression cassette for α-amylase. This cassette has an α-leader sequence and an α-amylase gene from Aspergillus oryzae (58). As a control, an empty CPOT plasmid was used. The *GCN2* gene was disrupted with the help of plasmid pECAS9-gRNA-kanMX, which contains both a *cas9* gene and a guide RNA (gRNA) expression cassette (59). The plasmids pECAS9-gRNA-kanMX-GCN2 and pECAS9-gRNA-kanMX-URA3 were made using pECAS9-gRNA-kanMX-tHFD1 as the template (59). First, the backbone was obtained by linearizing pECAS9-gRNA-kanMX-tHFD1 by digestion with MunI and EcoRI. The left fragment was constructed with primer 54 in combination with either 53 (*GCN2*) or 61 (*URA3*), and the right fragment was constructed with primer 55 in combination with either 52 (*GCN2*) or 60 (*URA3*). The correct assembly of the plasmids was confirmed by sequencing using primer 42. The genomic deletion was verified using primer pairs 38 and 39 for *GCN2* and 40 and 41 for *URA3*. An overview of the plasmids used in this study can be found in Table 1. The sequences of the primers used to make the gRNA and repair fragments and their verification can be found in Table 2. Escherichia coli DH5α was used for plasmid amplification.

**TABLE 1:** List of plasmids used in this study

Plasmid	Description	Reference or source
pAlphaAmyCPOT	2μ vector with cassette expressing POT1 gene from S. pombe and an expression cassette with α-leader sequence and α-amylase gene under native TPI1 promoter and terminator	58
pCPOT	2μ vector with cassette expressing POT1 gene from S. pombe	58
pECAS9-gRNA-kanMX-tHFD1	2μ vector with kanMX marker expressing eCas9 under the TEF1 promoter and CYC1 terminator and the gRNA targeting HDF1 under the SNR52 promoter	59
pECAS9-gRNA-kanMX-tGCN2	2μ vector with kanMX marker expressing eCas9 under the TEF1 promoter and CYC1 terminator and the gRNA targeting GCN2 under the SNR52 promoter	This study
pECAS9-gRNA-kanMX-tURA3	2μ vector with kanMX marker expressing eCas9 under the TEF1 promoter and CYC1 terminator and the gRNA targeting URA3 under the SNR52 promoter	This study
pRS416TEF roGFP2-PRX1	2μ vector with URA3 marker with a expression cassette with roGFP2-PRX1 under the TEF1 promoter and CYC1 terminator	29
pRS416TEF roGFP2-PfAOP	2μ vector with URA3 marker with a expression cassette with roGFP2-PfAOP under the TEF1 promoter and CYC1 terminator	33
pRS416TEF roGFP2-PfAOP409M	2μ vector with URA3 marker with a expression cassette with roGFP2-PfAOP409M under the TEF1 promoter and CYC1 terminator	33
pVW31	2μ vector with URA3 marker with a firefly luciferase cDNA under control of a fragment of the GCN4 promoter and an independent cassette with renilla luciferase under control of native PGK1 promoter	39

**TABLE 2:** List of primers used for strain construction in this study

Primer	Description^*a*^	Template	Sequence
54	Fw; EcoRI cut site	pECAS9-gRNA-kanMX	GGAACAACACAAACACTAC
55	Rv; Munl cut site	pECAS9-gRNA-kanMX	CAAAGGAAATGATAGCATTGAA
52	Fw; GCN2 gRNA	pECAS9-gRNA-kanMX	ATAAATGATCAATGTTATAGAAGATTCAACGTTTTAGAGCTAGAAATAGCAAG
53	Rv; GCN2 gRNA	pECAS9-gRNA-kanMX	GCTCTAAAACGTTGAATCTTCTATAACATTGATCATTTATCTTTCACTGCG
60	Fw; URA3 gRNA	pECAS9-gRNA-kanMX	ATAAATGATCGGGTCAACAGTATAGAACCGGTTTTAGAGCTAGAAATAGCAAG
61	Rv; URA3 gRNA	pECAS9-gRNA-kanMX	GCTCTAAAACCGGTTCTATACTGTTGACCCGATCATTTATCTTTCACTGCG
38	Fw; GCN2 genomic verification	Chromosome IV	GCCTCACACAACATACGCAC
39	Rv; GCN2 genomic verification	Chromosome IV	GGAGGAAGCAGTCACCCATC
40	Fw; URA3 genomic verification	Chromosome V	ACGAAGGAAGGAGCACAGAC
41	Rv; URA3 genomic verification	Chromosome V	CCAGTACACCTTATCGGCCC
42	Fw; upstream gRNA on the Cas9 plasmid	pECAS9-gRNA-kanMX	GGACGCTCGAAGGCTTTAAT

a Fw, forward; Rv, reverse.

### Media and culture conditions.

Media used for S. cerevisiae strain construction were YPD, YPE, YPEG, and SD-URA. The experiments were always performed at 30°C and 220 rpm. YPD medium contained 10 g/liter yeast extract, 20 g/liter peptone, and 20 g/liter glucose and was used for all cultures unless otherwise mentioned. For the selection of the kanMX marker on the CRISPR plasmid, 200 mg/liter G418 (Formedium, Hunstanton, UK) was added to the YPD medium. The YPE medium contained 10 g/liter yeast extract, 20 g/liter peptone, 20 g/liter absolute ethanol and was used solely as a solid medium. For liquid cultivations, 30 g/liter glycerol was added to YPE, and the medium was referred to as YPEG. Both YPE and YPEG were used only for S. cerevisiae strains without CPOT plasmids, since those are unable to ferment glucose as the sole carbon source (60). SD-URA contained 20 g/liter glucose, 6.7 g/liter yeast nitrogen base without amino acids, and 0.77 g/liter complete supplement mixture without uracil (CSM-URA; Formedium) This medium was only used to verify the deletion of the *URA3* gene. To solidify media, 20 g/liter agar (Merck Millipore) was added. The protein expression and physiological experiments were performed in SD2XSCAA medium with glutamine instead of glutamate. SD-2XSCAA medium contained 20 g/liter glucose, 6.9 g/liter yeast nitrogen base without amino acids, 190 mg/liter Arg, 400 mg/liter Asp, 1260 mg/liter Gln, 130 mg/liter Gly, 140 mg/liter His, 290 mg/liter Ile, 400 mg/liter Leu, 440 mg/liter Lys, 108 mg/liter Met, 200 mg/liter Phe, 220 mg/liter Thr, 40 mg/liter Trp, 52 mg/liter Tyr, 380 mg/liter Val, 1 g/liter BSA, 5.4 g/liter Na_2_HPO_4_, and 8.56 g/liter NaH_2_PO_4_·H_2_O and had a pH of 6.4.

Characterization of the roGFP2 sensors was performed in Delft synthetic medium (61), and the verification of the luciferase expression was done in defined synthetic medium lacking uracil, using 14-ml cultivation tubes (62). Protein production experiments and *GCN4* expression experiments were performed at 30°C at 220 rpm in aerated 24-well plates (CR1224; Bioscreen) with a volume of 2.5 ml and a starting optical density at 600 nm (OD_600_) of 0.01. All other samples were grown in 100-ml shake flasks with 10 ml SD2XSCAA medium and a starting OD_600_ of 0.01. The cultures for qPCR analysis were grown in a volume of 20 ml with a starting OD_600_ of 0.01. E. coli cells were grown in Luria-Bertani (LB) medium at 37°C and 200 rpm. Selection medium contained 80 mg/liter ampicillin. The transformation procedure used for E. coli was according to a known protocol (63).

### Molecular biology techniques.

S. cerevisiae strains were transformed according to the protocol using the Li/Ac SS carrier method (64). Five hundred nanograms of DNA was used for the transformation of plasmids and an additional 1 μg repair fragment when required. To verify deletions or test for the presence of the CPOT plasmids, colony PCR was performed using SapphireAmp fast PCR mix (TaKaRa Bio). For DNA construction, Phusion high-fidelity DNA polymerase (Thermo Scientific) was used. Restriction digestion was performed using FastDigest (Thermo Scientific) products. All techniques were used according to the manufacturer’s protocols unless otherwise stated.

### α-Amylase assay.

Cells were harvested after 24 h, 48 h, and 96 h. Cells were pelleted by centrifugation at 4°C, 8,000 rpm for 5 min, and then the supernatant was used for the α-amylase quantification assay. The Ceralpha kit (Megazyme) was used with α-amylase from Aspergillus oryzae as the standard. The assay was performed according to the manufacturer’s protocol, with the exception of the preparation of buffer A. Since the protein was dissolved in the medium, instead of preparing buffer A and dissolving solidified protein, we used a mixture of medium and Milli Q water, depending on the concentration of α-amylase, to make buffer A with the correct concentration and protein. We used a dilution of 200× or 400× depending on the concentration of α-amylase in the medium.

### Growth profiler.

The S. cerevisiae strains were cultivated for 48 h in 250 μl SD2XSCAA medium at 30°C and 1,200 rpm in 96-well plates (CR1496d; Enzyscreen). Growth curves were measured using a Growth Profiler 960 (Enzyscreen). Three independent colonies per strain were grown in 1 ml SD2XSCAA medium in 7-ml cultivation tubes after an overnight culture. The cells were then inoculated in technical triplicates with a starting OD_600_ of 0.005.

### Microbioreactor cultures.

S. cerevisiae strains were cultivated for 96 h in 1 ml SD2XSCAA medium at 30°C and 1,200 rpm in flower plates. The characterization of the sensors was performed in Delft minimal medium and the experiments in SD2XSCAA medium. Three independent colonies per strain were grown in 1 ml SD2XSCAA medium in 7-ml cultivation tubes after an overnight culture. Cells were then inoculated in technical duplicates with a starting OD_600_ of 0.005. For measuring the biomass, excitation and emission at 600 nm was used with a gain of 20; for the oxidation of cysteine, excitation at 405 nm and emission at 520 nm with a gain of 100 were used; and for the reduction of cysteine, excitation at 488 nm and emission at 520 nm with a gain of 100 were used. All wells were measured every 20 min by a Biolector microbioreactor system (M2p-Labs).

### Ox/Red ratio determination.

Background fluorescence was determined using strains carrying an empty p416 vector. We used biological duplicates of these controls with technical duplicates. The natural fluorescence per strain was determined at both 405 nm (Ox) and 488 nm (Red). For both wavelengths, the average natural fluorescence was determined. These average values were subtracted from the Ox and Red measurements of all the separate replicates with the roGFP2 sensors. The GFP signals with the natural fluorescence subtracted were used to determine the Ox/Red ratio per replicate per strain. The final Ox/Red ratio was determined by taking the average of the ratios per strain. R Studio software was used for all data analyses (65).

### qPCR.

Cells were harvested after 24 h and 48 h, and cells were then instantly cooled on ice and centrifuged at 4°C, 6,000 rpm, for 3 min. The supernatant was discarded, and the pellet was snap-frozen using liquid nitrogen. For RNA extraction, the RNeasy kit (Qiagen) was used according to the manufacturer’s protocol. For cDNA synthesis, the Quantitect reverse transcriptase kit (Qiagen) was used. For the qPCR, the DyNaMo ColorFlash SYBR green qPCR kit was used. All primers used for qPCR are listed in Table 3 and were verified using the MIQE guidelines, with *ACT1* used as the reference gene.

**TABLE 3:** List of primers used for qPCR in this study

Direction	Gene	Sequence
Fw	HAC1 spliced	GCGTCGGACCAAGAGACTTC
Rv	HAC1 spliced	CTGACTGCGCTTCTGGATTAC
Fw	HAC1 unspliced	CAATTGGCGTAATCCAGCCG
Rv	HAC1 unspliced	AGCTGGGGCTAGTGTTCTTG
Fw	ERO1	CGCTTGCTCTGTTGATGTCG
Rv	ERO1	TCGTCATCGCTATCATCCGC
Fw	PDI1	CCCAGGTGGTAAGAAGTCCG
Rv	PDI1	CAATTCAGCGTCAGCATCGG
Fw	CTT1	GCTTCTCAATACTCAAGACCAG
Rv	CTT1	GCGGCGTATGTAATATCACTC
Fw	fRMsr	GTCATTGATCTGGCATGCGT
Rv	fRMsr	CCGCAAACACCTTTACCGAA
Fw	TSA1	ACACCAACCACTCTTTGTCCAG
Rv	TSA1	GGCTTCAACCAATCTCAAGGCTTC
Fw	TRX2	GAACAATATTCTGACGCTGCT
Rv	TRX2	CCGACGACTCTGGTAACCT
Fw	SRX1	CCAACAGAAATTCCTCTCTCA
Rv	SRX1	AGCCGCCTCAGCCTGTTC
Fw	EUG1	ATGGTCAAGTCTATCGCGGT
Rv	EUG1	TTCAAGCCTGTCAAGCCTCT
Fw	KAR2	TCAATGACGCGCAAAGACAA
Rv	KAr2	TCGAAAGTACCACCACCCAA
Fw	SCJ1	TTGTCAAGGTCGTGGGGTTA
Rv	SCJ1	ATGTAGTTTCTTGGTGCGCC
Fw	LHS1	GGACACTACTCAGCCCGTTA
Rv	LHS1	TTTGCCTTCTCTGCTGCTTG
Fw	JEM1	ACGTTCAACTGTCGAGACCT
Rv	JEM1	CTCTTGACGTCCGGACTCAT
Fw	FKB2	GGCTCTCCTATCGCTTTTGA
Rv	FKB2	CAGCACTTGGAGGAATGACG

### Puromycin treatment.

Yeast cells were grown in SD2XSCAA and grown until the mid-exponential phase (OD_600_ of ∼1). Cells were then normalized to an OD_600_ of 1 and then harvested and collected by centrifugation before being incubated in 100 ml phosphate-buffered saline (PBS) with 1 mM puromycin for 10 min at 30°C, 220 rpm. Cells were then collected by centrifugation, and intracellular proteins were extracted as described previously (66). Ten microliters of the cell extracts was then used for SDS-PAGE and Western blot analysis.

### eIF2α protein extraction.

For the intracellular protein extraction of the elongation factor eIF2α, protein extraction with LiAc/NaOH was performed as in reference 67. Yeast cells were harvested after 24 h and 48 h (OD_600_ of 5) and at 72 h and 96 h (OD_600_ of 10), and 10 μl of the cell extracts was used for SDS-PAGE and Western blot analysis.

### Western blotting.

Samples and controls were loaded and separated with stain-free 4 to 20% gels (Bio-Rad). Proteins were transferred onto 0.45-μm polyvinylidene difluoride membranes (Bio-Rad) using the Trans-blot turbo transfer system (Bio-Rad). The blot was blocked using Western blocker solution (Sigma-Aldrich) and incubated in either anti-total eIF2α (1:1,000), anti-puromycin (1:1,000), or eIF2α-phosphorylated (1:1,000; Ser-51; Invitrogen), followed by incubation with either anti-mouse (1:5,000) or anti-rabbit (1:5,000). Both secondary antibodies were horseradish peroxidase (HRP) conjugated, visualized using West Pico plus HRP substrate (Thermo Fischer), and measured with a ChemidoC XRS image analyzer (Bio-Rad).

### Viability measurements.

Cell viability was measured using propidium iodide (Invitrogen) staining as described previously (66). Samples were taken after 1, 2, 3, 4, and 13 days of cultivation in 10 ml of SD2XSCAA medium from 100-ml shakeflasks. Fluorescence was measured with a Guava easyCyte 8HT system (Merck Millipore). For each sample, 5,000 cells were counted. The cultivations were performed in biological triplicate, and unstained cells were used as a negative control for the fluorescence measurements.

### *GCN4* expression assay.

The luciferase construct was tested in an S. cerevisiae BY4742 strain in which the pVW31 plasmid was transformed. Three biological replicas were cultivated in 7 ml cultivation tubes to which 10 mM (final concentration) 3-AT was added and incubated for 30 min. Luminescence was checked before and after the addition of 3-AT (3-aminotriazole). For the *GCN4* expression experiment, cells were harvested after 24 h and 48 h, and 2 ml of culture was centrifuged for 5 min at 35,000 rpm at 4°C. The supernatant was then discarded, and cells were washed in 1 ml cold water. Cells were resuspended in 300 μl PBS buffer with protease inhibitors and added to lysin matrix tubes (MP Bio). The mixture was Fast prepped at 5,000 rpm for 20 s 3 times, with incubation of the samples on ice between runs. The mixture was then centrifuged for 10 min at maximum speed at 4°C, and 100 μl of clear supernatant was harvested and stored at −20°C. Luminescence was measured with a FluoStar Omega plate reader (BMG Labtechnologies) and treated with the protocol and reagents of the Dual-Luciferase reporter assay system (Promega). All reagents were used according to the manufacturer’s protocol.
