# Supplementary material for: The Yeast eIF2 Kinase Gcn2 Facilitates H2O2-Mediated Feedback Inhibition of Both Protein Synthesis and Endoplasmic Reticulum Oxidative Folding during Recombinant Protein Production
Source: Appl Environ Microbiol. 2021 Jul 13;87(15):e00301-21. doi: 10.1128/AEM.00301-21 (PMC8276805; doi:10.1128/AEM.00301-21)
Supplement: Supplemental file 1 — Figures S1 to S8. Download AEM.00301-21-s0001.pdf, PDF file, 1.0 MB [file aem.00301-21-s0001.pdf]

Supplementary Figures.

Supplementary Figure 1

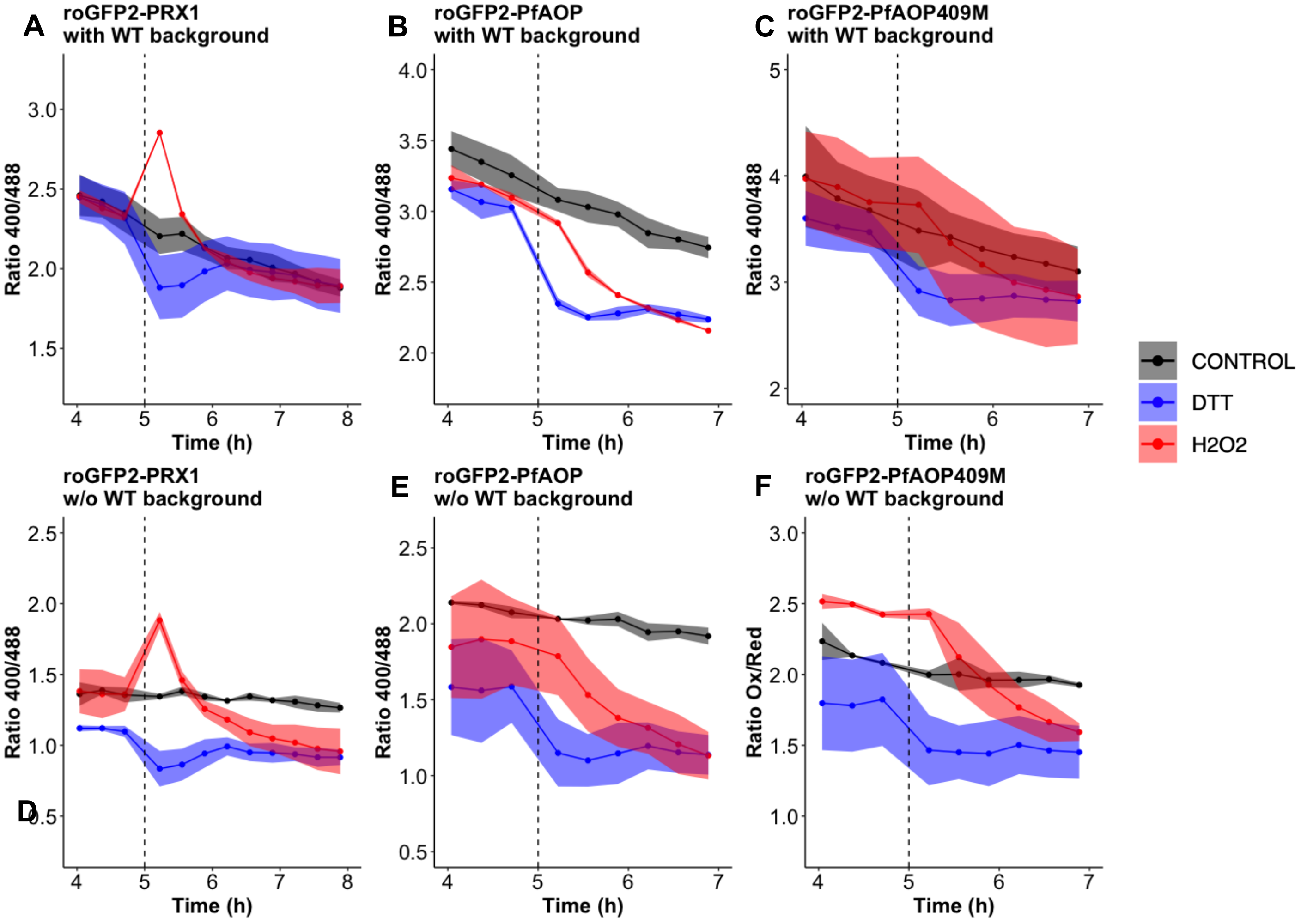

Figure S1. Verification of response of roGFP2 sensors to external additions of DTT and H_2O_2. GFP400/GFP488 ratio levels of cultivation with CEN.PK strains with plasmid-based expression of roGFP2-PRX1 (A&D), roGFP2-PfAOP (B&E) and roGFP2-PfAOP409M (C&F) grown in Delft synthetic medium. DTT and H_2O_2 were added to a final concentration of 1 mM after 5 h of cultivation. Plot A/B/C show the data without correction for the natural fluorescence and D/E/F have been corrected for the natural fluorescence. Cultivation with 1mM addition of DTT (blue), cultivation with 1 mM addition of H_2O_2 (red) and the control without addition (black). The data are based on biological duplicates, the solid line shows the average and the transparent parts the standard deviation.

Supplementary Figure 2.

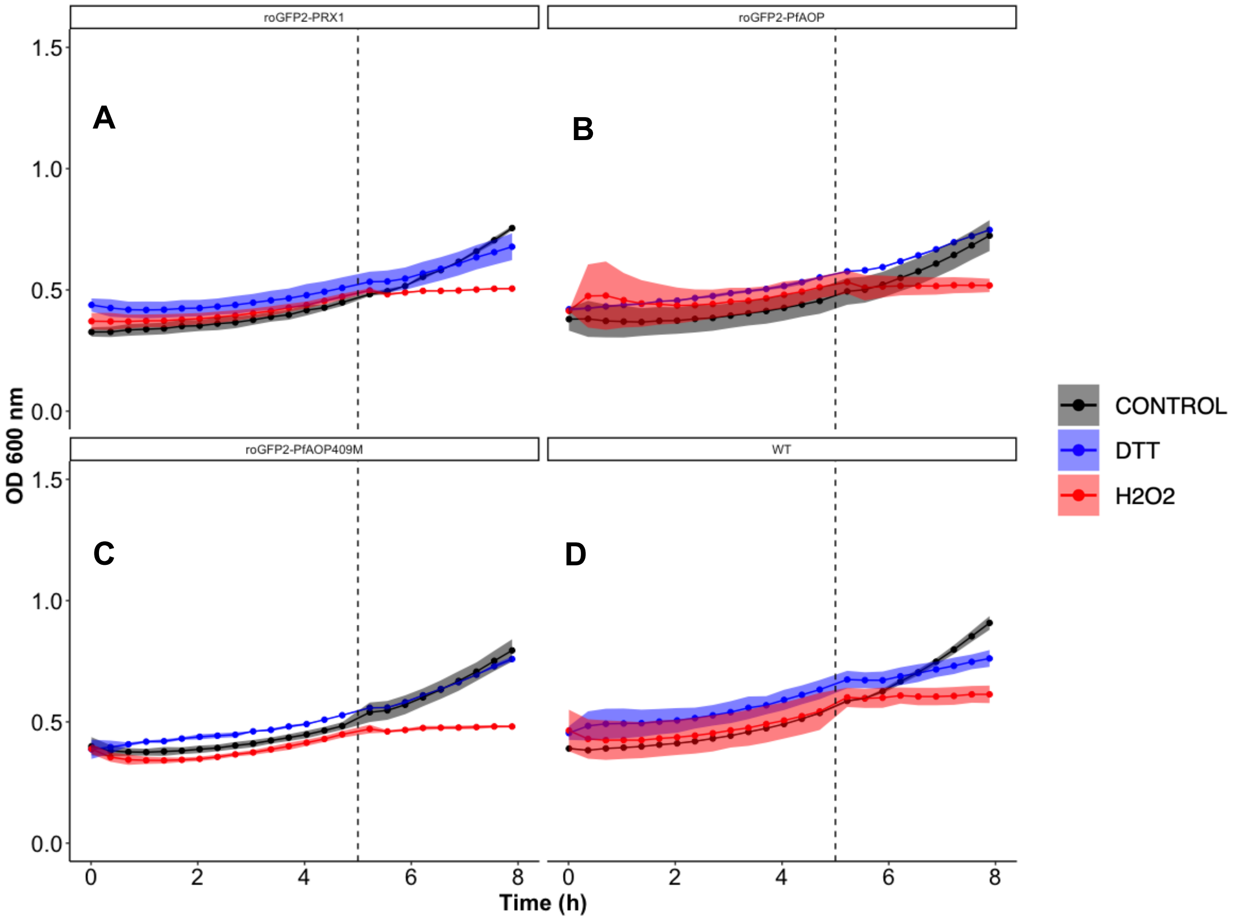

Figure S2. Growth curves of the CEN.PK strains with the roGFP2 sensors during treatment with DTT and H_2O_2.

OD_{600} of cultivation with CEN.PK strains with plasmid-based expression of roGFP2-PRX1 (A), roGFP2-PfAOP (B) and roGFP2-PfAOP409M (C) grown and the control with an empty vector (D) in Delft synthetic medium. DTT and H_2O_2 were added to a final concentration of 1 mM after 5 h of cultivation. Cultivation with 1mM addition of DTT (blue), cultivation with 1 mM addition of H_2O_2 (red) and the control without addition (black). The data are based on biological duplicates and the light colors represents the standard deviation.

Supplementary Figure 3

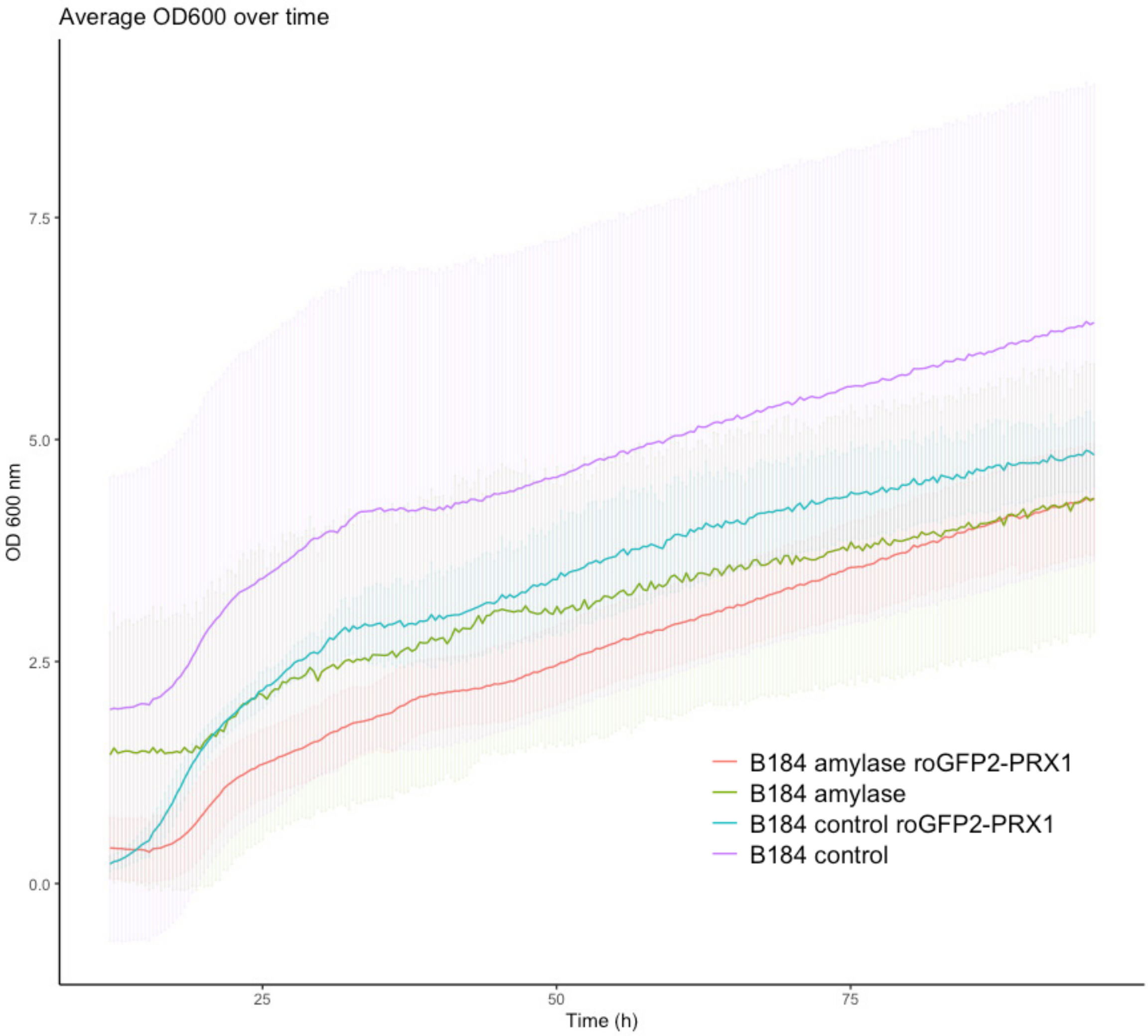

Figure S3. Biomass growth during microbioreactor cultivation of B184 with the roGFP2-sensor and the control.
Biomass growth over 96 h of cultivation for B184 with the roGFP2 plasmid or a plasmid control cultivated in SD2xSCAA medium. B184 expressing α-amylase and roGFP2 (red), B184 expressing α-amylase without expressing roGFP2 (green), B184 control without expressing α-amylase with expressing roGFP2 (blue) and B184 control without expressing α-amylase and roGFP2 (purple). The light bars represent the standard deviations of three biological replicas and two technical replicas. The first 15 h were excluded due to too low signal.

Supplementary Figure 4

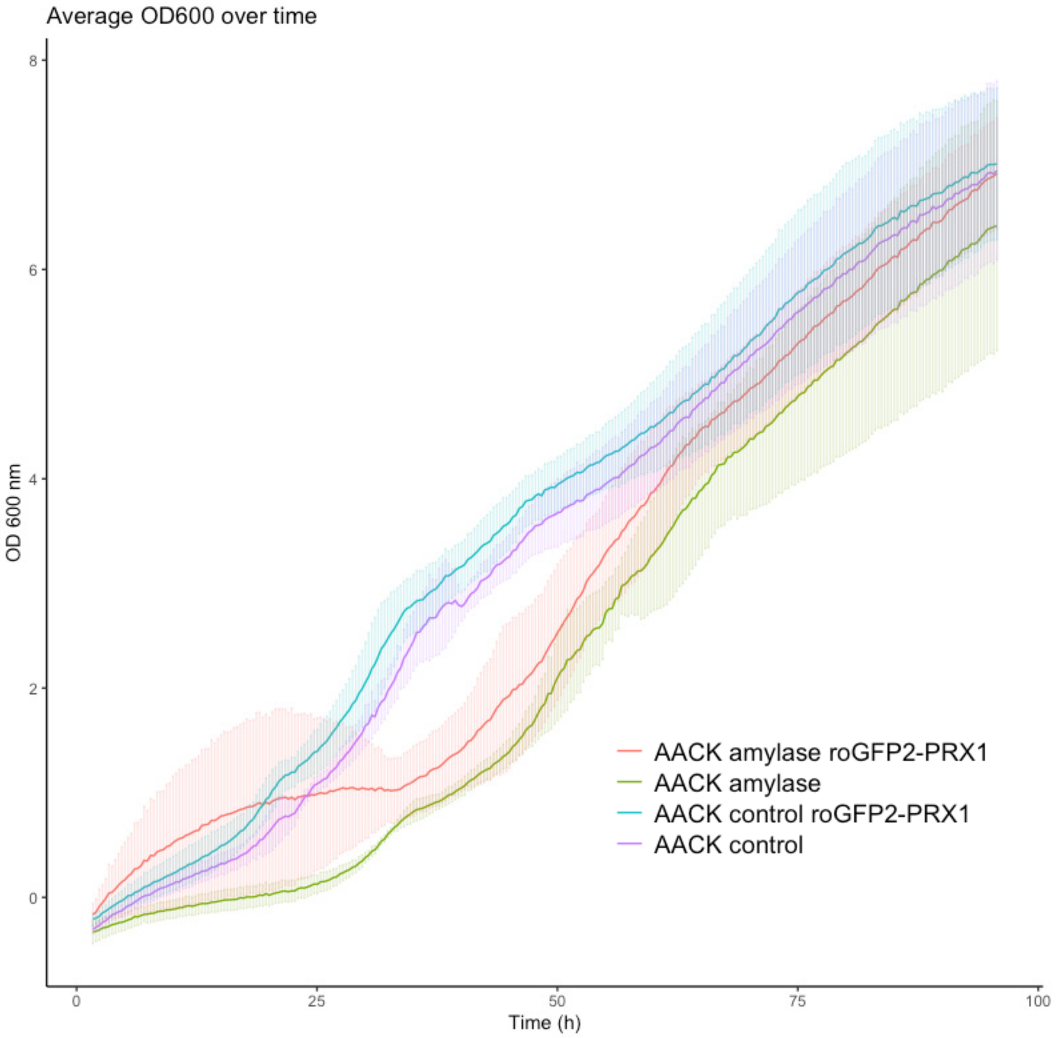

Figure S4. Biomass growth during microbioreactor cultivation of AACK with the roGFP2-sensor and the control.
Biomass growth over 96 h of cultivation for AACK with the roGFP2 plasmid or a plasmid control cultivated in SD2xSCAA medium. AACK expressing α-amylase and roGFP2 (red), AACK expressing α-amylase without expressing roGFP2 (green), AACK control without expressing α-amylase with expressing roGFP2 (blue) and AACK control without expressing α-amylase and roGFP2 (purple). The light bars represent the standard deviations of three biological replicas and two technical replicas. The first 15 h were excluded due to too low signal.

Supplementary Figure 5

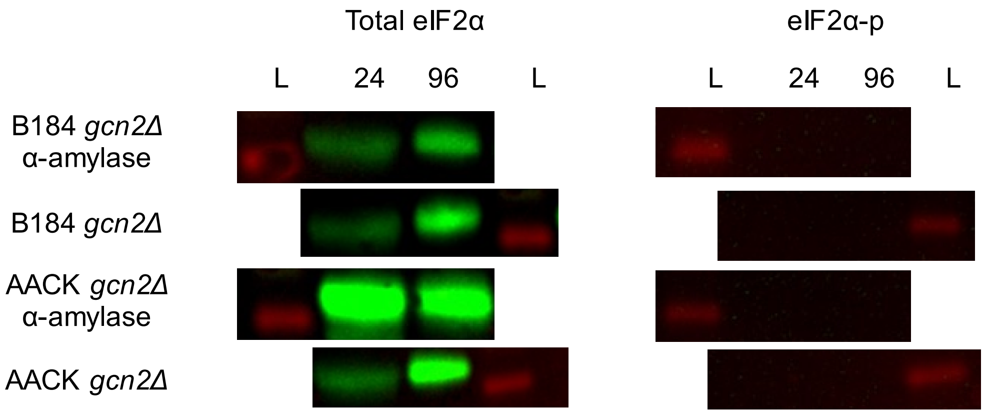

Figure S5. Western blot data of the total eIF2a and eIF2a-phosphorylated of AACK and B184 *gcn2Δ.*

Western blot of total eIF2a and eIF2a-phosphorylated during cultivation after 24 h and 96 h of AACK *gcn2Δ* and B184 *gcn2Δ* with and without producing α-amylase. The L indicated the lanes with the protein ladder

Supplementary Figure 6

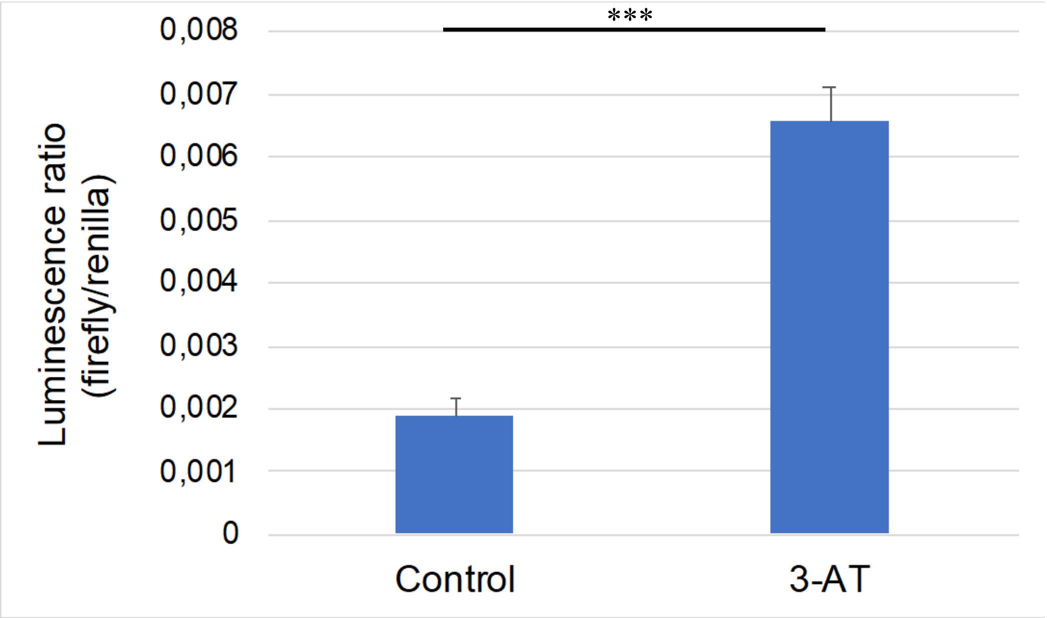

Figure S6. Translation of the *GCN4* luciferase construct increases upon chemical amino-acid starvation (3-AT).
Luminescence ratio of the Firefly/Renilla luminescence before and after induction with 10 mM 3-AT. The control samples were harvested before the addition of 3-AT and the 3-AT samples after a 30 min incubation period. The luminescence was determined for the firefly luciferase indicating *GCN4* expression and the renilla luciferase the plasmid abundancy. The data are based on three biological replicates and errors bars show the standard deviations.

Supplementary Figure 7.

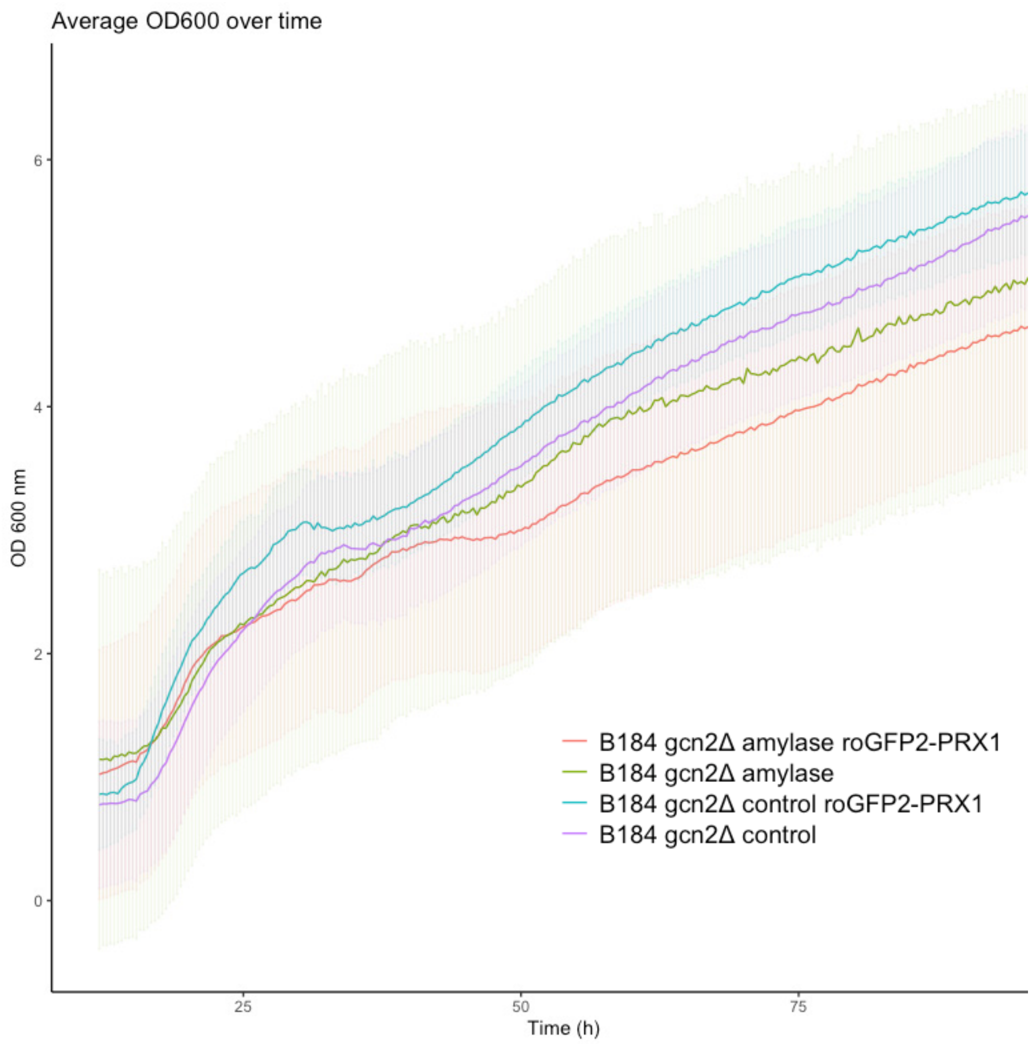

Figure S7. Biomass growth during microbioreactor cultivation of B184 *gcn2Δ* with the roGFP2-sensor and the control.

Biomass growth over 96 h of cultivation for B184 *gcn2Δ* with the roGFP2 plasmid or with a plasmid control cultivated in SD2xSCAA medium. B184 *gcn2Δ* expressing α-amylase and roGFP2 (red), B184 *gcn2Δ* expressing α-amylase without expressing roGFP2 (green), B184 *gcn2Δ* control without expressing α-amylase with expressing roGFP2 (blue) and B184 *gcn2Δ* control without expressing α-amylase and roGFP2 (purple). The light bars represent the standard deviations of three biological replicas and two technical replicas. The first 15 h were excluded due to too low signal.

Supplementary Figure 8.

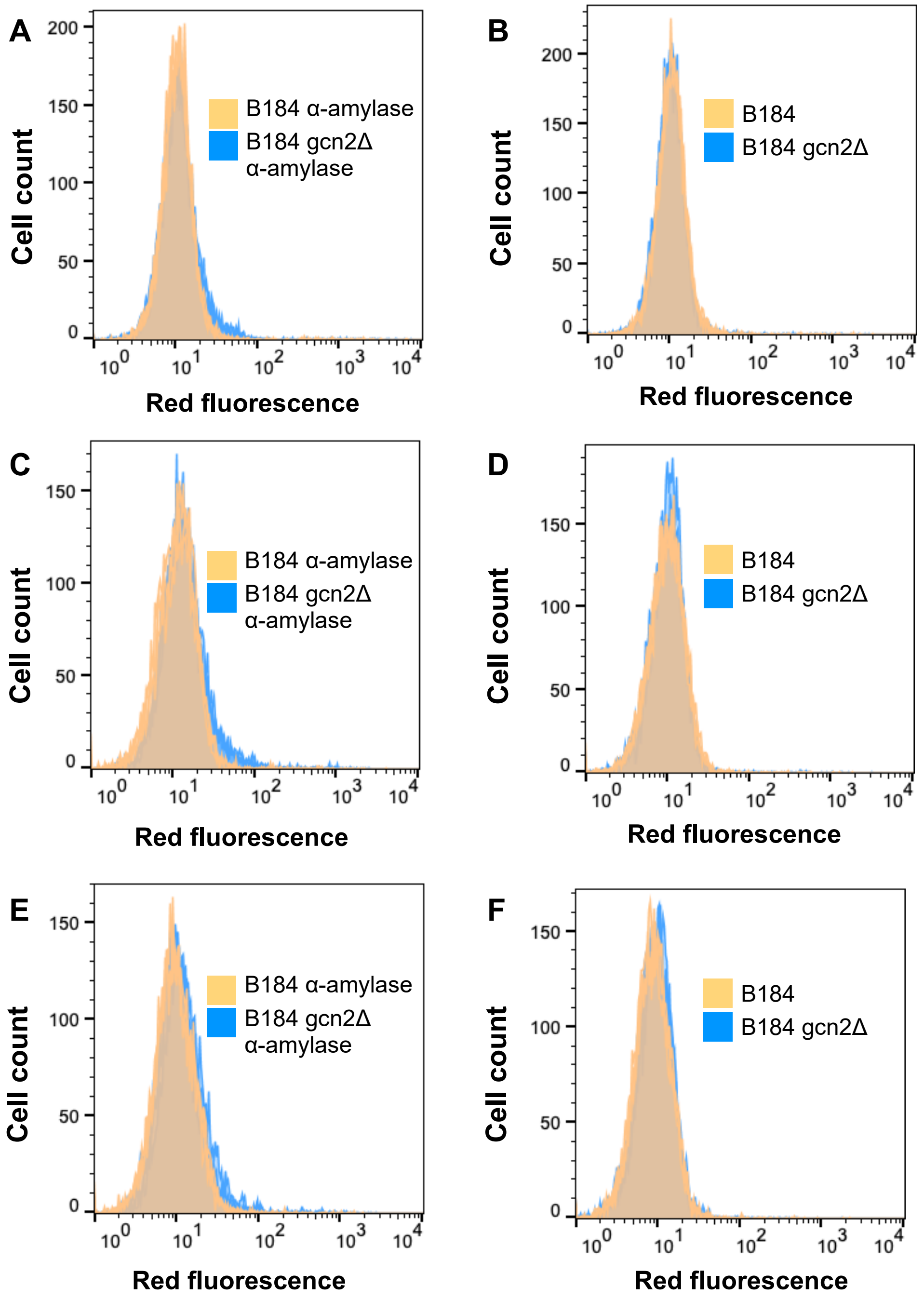

Figure S8. Viability of B184 and B184 *gcn2Δ* with and without recombinant protein production during 96 h of cultivation.

Flow cytometry histograms with B184 (orange) and B184 *gcn2Δ* (blue) expressing recombinant α-amylase stained with PI staining. (A) B184 and B184 *gcn2Δ* expressing recombinant α-amylase after 24 h (B) B184 and B184 *gcn2Δ* without expression of a recombinant protein after 24 h, (C) B184 and B184 *gcn2Δ* expressing recombinant α-amylase after 48 h (D) B184 and B184 *gcn2Δ* without expression of a recombinant protein after 48 h, (E) B184 and B184 *gcn2Δ* expressing recombinant α-amylase after 96 h and (F) B184 and B184 *gcn2Δ* without expression of a recombinant protein after 96 h.
